# Internal jugular access using pocket ultrasound in a simulated model: comparison between biplane and monoplane visualization techniques

**DOI:** 10.1186/s13089-023-00335-4

**Published:** 2023-10-10

**Authors:** Jair Antonio Ruiz Garzón, Gloria Catalina Zuluaga López, Laura B. Piñeros-Hernandez, Yury Forlan Bustos Martínez

**Affiliations:** 1 Advanced Center for Clinical Simulation and Technological Innovation, School of Medicine and Health Sciences, Universidad del Rosario, Bogotá, Colombia; 2 Universidad del Rosario, Bogotá, Colombia

**Keywords:** Vascular access, Point of care ultrasound, Biplane, Catheterization complication, Simulation

## Abstract

**Introduction:**

Ultrasound is the current standard for central venous access due to its advantages in efficiency and safety. In-plane and out-of-plane visualization techniques are commonly used, but there is no clear evidence showing an advantage of one technique over the other. The objective of this study was to compare the success and time required for biplane visualization vs. in-plane and out-of-plane techniques in simulated models.

**Methodology:**

Ten emergency medicine specialists participated in 60 simulated events, with randomization of the visualization technique for each event. Each event required intravenous cannulation of a simulated model for jugular venous access, with a maximum of three attempts allowed. The number of attempts required for each event, success of puncture and venous cannulation, frequency of redirection and puncture of the posterior wall, time required to obtain an optimal window, visualize the needle inside the vessel, and passage of the guidewire were recorded. The success ratios and times required for each visualization technique (biplane, in-plane, and out-of-plane) were compared.

**Results:**

Cannulation success rate was 100% for all three techniques. Success on the first attempt was 95% for biplane visualization vs. 100% for in-plane and out-of-plane. The median total time for the procedure was higher for biplane visualization (29.9 s) compared to in-plane (25.2 s) and out-of-plane (29 s), but this difference was not statistically significant (*p* = 0.999). There were no significant differences in cannulation success, needle redirection, or posterior wall puncture frequency between biplane visualization and in-plane and out-of-plane techniques.

**Conclusions:**

This study suggests that biplane visualization with the use of pocket ultrasound for internal jugular cannulation in simulated models did not demonstrate significant differences when compared with in-plane and out-of-plane visualization techniques. Further research with larger sample sizes may be needed to confirm these results.

## Introduction

Central venous access is a common procedure used for patients both inside and outside of intensive care units to insert various devices for patient hemodynamic monitoring, pharmacological and hydroelectrolytic therapies, renal replacement therapies, parenteral nutrition, and cardiac stimulation, among others [1]. In the United States, more than 5 million central venous catheters are inserted each year [2, 3]. Central catheter use is higher in the ICU (55.4%) than outside the ICU (24.4%) [4], representing a significant cost to the healthcare system of 17–29 billion dollars per year [5].

This intervention is associated with thrombotic, infectious, and mechanical complications, with reported frequencies of 15–26% [3, 6]. However, the use of ultrasound as a guide for insertion visualization has led to a decrease in complication rates in recent years. Systematic reviews have shown a decrease in complication rates of up to 71% with the use of ultrasound [7], resulting in a higher success rate and a complication rate of 4.6% [8]. Major medical associations now recommend its use [9–12].

The in-plane and out-of-plane techniques are the most frequent techniques used for ultrasound-guided central venous access. However, no difference has been found between them [13–15]. Recently, the biplane technique has been reported as another technique that combines the two techniques mentioned above for the passage of central catheters [16–18]. This study aims to compare the biplane technique vs. in-plane and out-of-plane techniques for the insertion of jugular central venous accesses using a pocket ultrasound machine.

## Methodology

This is a single-factor experimental study conducted in simulation with a quantitative approach, developed at the Advanced Center for Clinical Simulation and Technological Innovation of the Universidad del Rosario in Bogota, Colombia. Specialists in emergency medicine with experience in ultrasound-guided vascular access were recruited to perform the punctures using visualization techniques in one and two planes. The call was kept open until 10 specialists who met the inclusion criteria were recruited and subsequently agreed to participate under informed consent. Based on previous studies, cannulation success in the first attempt was estimated at 90% for the biplane technique and 50% for the in-plane and out-of-plane techniques [1], requiring 60 procedures (20 for each technique) to achieve a power of 80% and an α error of 0.05, allowing detection of the difference between the ultrasound windows and the outcomes under study. The arcosine approximation was used, employing the Granmo application version 7.12 April 2012.

Prior to each set of three events, a random assignment of the three ultrasound planes was conducted to determine which one would be used. The randomization was performed using the statistical package R Core Team 4.2.2.

**Fig. 1:**
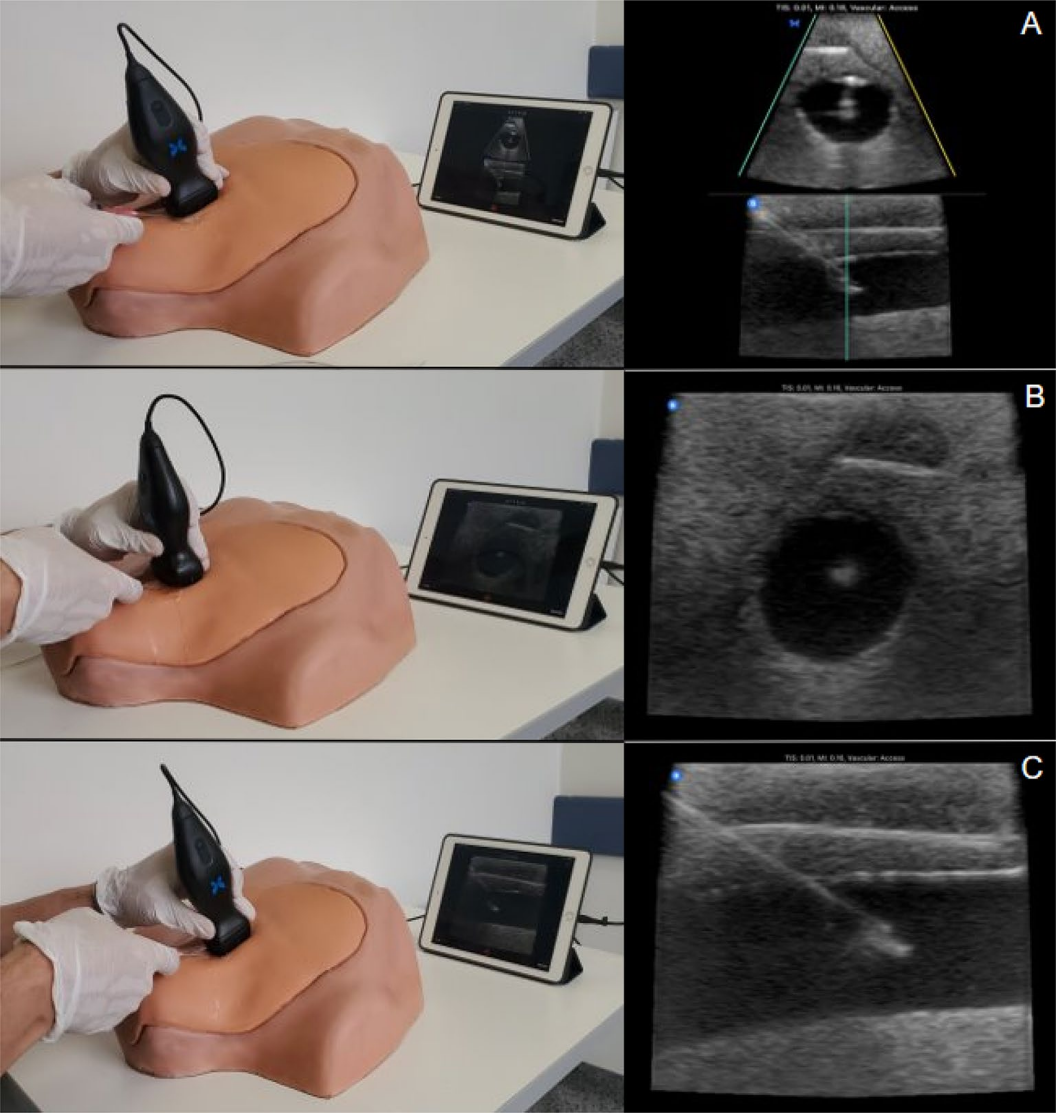
Ultrasound planes for vascular access. **A** Biplane, **B** Out-of-plane, **C** In-plane

The procedure was performed on a simulated model for central vascular accesses, CentraLineMan^®^ System CML 50, which had external anatomical landmarks include clavicle, sternal notch, sternal and clavicular heads of the sternocleidomastoid muscle, manubrium, and lateral border of the first rib. The model was placed at a distance of 1.2 cm from the skin to the vessel at the lower third of the neck. A right jugular vein with a diameter of 1 cm and a right carotid artery located 7 mm away from the vein were used. The procedure utilized an adult central venous access device, Certofix^®^ Duo S 720, with an 18G × 2 ¾" needle, 0.89 mm diameter guide, and a length of 50 cm (Fig. 1).

Sociodemographic variables such as age and sex, experience-related variables, and procedure-related variables (time to window, time for internal jugular vein puncture, puncture success, posterior wall puncture, puncture success, redirection, and the number of punctures) were included.

The study was conducted in a controlled environment at a university simulation center. To ensure that participants were able to familiarize themselves with the ultrasound machine without becoming familiar with the characteristics of the actual target vessel, a different simulated model of the central venous access (Blue PhantomTM Central Venous Access) was used during the 15-min equipment familiarization period. This was done prior to the procedure, after each participant received a 6-min video explanation of the in-plane, out-of-plane, and biplane central venous access techniques. A researcher was present during this time to clarify any doubts about the operation of the ultrasound machine.

To perform the ultrasound windows, the Butterfly iQ + (pocket ultrasound scanner, reference 900-20006-01) was connected to an iPad Pro (9.7 inches) using the Butterfly Network V2.12.1 software, configured in the vascular access preset, with a depth of 3 cm and a gain of 90%. The participant could make adjustments considered pertinent to obtain real-time visualization of the best ultrasound window of the vessel to be cannulated.

The data were recorded immediately and stored digitally on university servers provided for this purpose (SharePoint-Office 365 license Universidad del Rosario), which only researchers had access to.

### Analysis

Categorical variables, such as gender, ultrasound plane, puncture success, cannulation success, redirection, and posterior wall puncture, were described using absolute and relative frequencies. Continuous variables, such as age, years of experience, specific experience, time to window, and time to vessel, were described using means and standard deviations or medians and interquartile ranges, depending on the distribution of the data. The normality of the data was explored using Shapiro–Wilk test.

To determine whether there were significant differences in proportions between the different ultrasound planes (biplane, in-plane, and out-of-plane), the success of cannulation, the success of puncture and posterior wall puncture, and the need to redirect the needle, Fisher's exact test was performed. To determine whether there were significant differences in medians (according to normality) between the different ultrasound planes and the time to window, time to vessel, and number of punctures, the Kruskal–Wallis test was applied. For cannulation time, the one-factor ANOVA test was used after checking the assumptions. Post hoc tests were used to further explore significant differences. A statistical significance level of 0.05% and a 95% confidence interval were used for all tests. The statistical analysis was performed using Jamovi software version 2.2.5.

## Results

Ten expert specialists in emergency medicine with experience in ultrasound-guided vascular access were included in the study. Each expert performed a total of six procedures, two for each ultrasound plane evaluated (biplane, in-plane, and out-of-plane). Eighty percent of participants were men, with a mean age of 36 years (RIQ 6.25) and a mean of specialist experience was 5 years (RIQ 2.5).

### Successful cannulation of the internal jugular vein

**Fig. 2:**
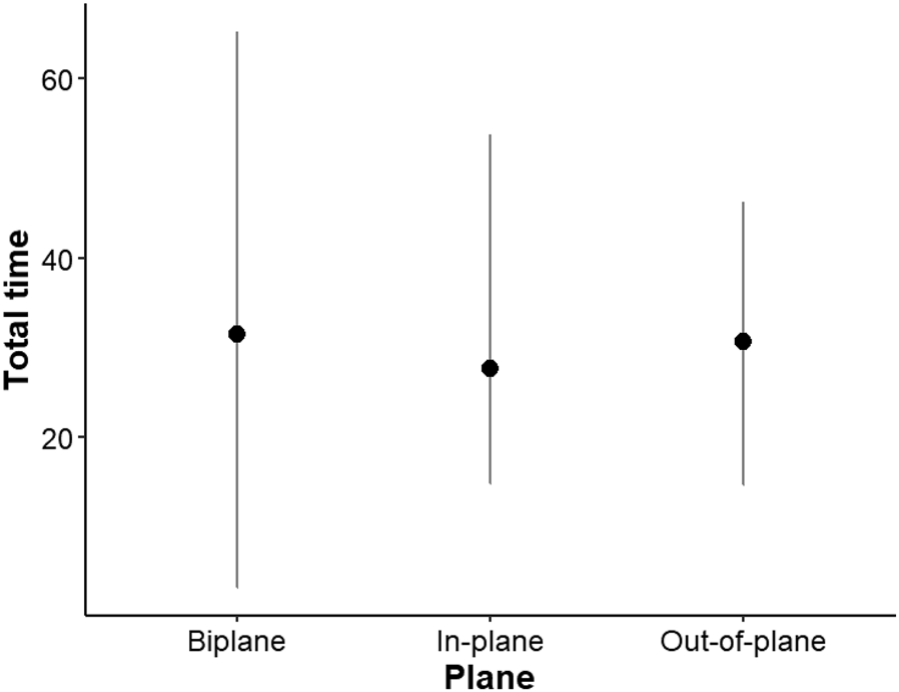
Time to cannulation by ultrasound imaging. The total time in seconds to internal jugular vein cannulation by ultrasound image was not significantly different between the three techniques (*p* = 0.458)

Successful cannulation of the internal jugular vein was achieved with all three techniques at 100%. The puncture success in the first attempt was 95% (*n* = 19/20) with biplane imaging and 100% (*n* = 20/20) with single-plane imaging (*p* = 0.362). The total time taken for each attempt was less with in-plane imaging (27.61 ± 8.96), followed by out-of-plane (30.63 ± 8.81) and biplane (31.45 ± 13.59; *p* = 0.327) (Fig. 2). No statistically significant differences were found when comparing the total procedure time, the time required to obtain the best ultrasound window, and the time required for puncture among the experts who performed the procedures.

Regarding the time required to obtain the correct ultrasound window of the internal jugular vein in the first attempt, it was shorter with the out-of-plane image (7.91 RIQ: 4.72), followed by biplane (7.13 RIQ: 4.13) and in-plane (7.25 RIQ: 5.75; *p* = 0.944), but no statistically significant differences were found (Table 1). The time taken from the puncture of the model surface to correct visualization of the needle inside the vessel was shorter in in-plane (5.49 RIQ: 5.49), followed by out-of-plane (6.23 RIQ: 6.23) and biplane (9.12 RIQ 9.55; *p* = 0.187). Puncture success was 100% with in-plane and out-of-plane images and 95% in biplane (*p* = 0.999).

**Table 1:** Results of outcomes according to ultrasound imaging

Variable		CI 95%	*p*
Time to window (s) (RIQ)	BP: 7.25 (5.75) IP: 7.91 (4.72) OP: 7.13 (4.13)	6.55–10.18 5.90–10.68 6.34–10.36	0.944^+^
Puncture success (%)	BP: 95% (19/20) IP: 100% (20/20) OP: 100% (20/20)		0.999^++^
Time for IJV puncture (s) (RIQ)	BP: 9.12 (9.55) IP: 5.49 (5.49) OP: 6.23 (6.23)	8.33–17.42 7.13–10.07 9.28–13.64	0.187^+^
Posterior wall puncture (%)	BP: 15% (3/20) IP: 10% (2/20) OP: 20% (4/20)		0.999^++^
Redirection (%)	BP: 25% (5/20) IP:15% (3/20) OP: 5% (1/20)		0.265^++^
Total time of the procedure (s)	BP: 31.45 ± 13.59 IP: 27.61 ± 8.96 OP: 30.63 ± 8.81	25.49–37.40 23.69–31.54 26.77–34.49	0.327^+^

*IJV* internal jugular vein, *BP* biplane, *IP* in-plane, *OP* out of plane, *IQR* interquartile range, *S*: time in seconds

^+^Kruskal–Wallis test. ^++^*X*^2^ and Fisher's exact test

There was only one failure in the first attempt when the puncture was not successful with the biplane technique. In the second attempt, the procedure was successful with a window time of 3.68 s, time to vessel of 15.74 s, and a total time of 29.89 s; there was no redirection or puncture of the posterior wall.

### Posterior wall puncture and redirection

**Fig. 3:**
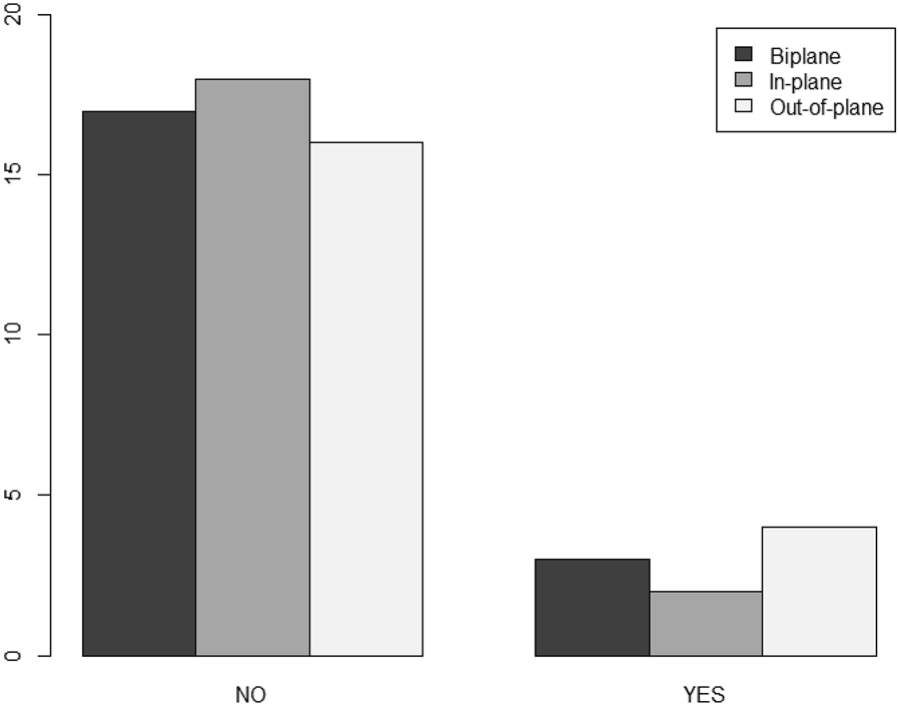
Puncture of the posterior wall according to ultrasound plane

With in-plane imaging, there were fewer punctures of the posterior wall during the procedure, followed by biplane and out-of-plane (10% vs. 15% vs. 20% *p* = 0.368), and redirections occurred more frequently in biplane, followed by in-plane and out-of-plane (25% vs. 15% vs. 5% *p* = 0.265) (Fig. 3).

## Discussion

According to the searches performed in different scientific databases by the researchers, this is the first study investigating the performance of a pocket ultrasound machine for internal jugular vein cannulation in a simulated model, comparing biplane visualization not only with out-of-plane visualization but also with in-plane visualization. The results indicate that contrary to the hypothesis of advantage with the biplane technique, no statistically significant differences were found in terms of success, execution time, frequency of posterior wall puncture, or need for needle redirection.

When comparing our study to the one conducted by Convissar et al., which used a simulated model for peripheral access with a smaller diameter compared to the larger diameter model used in our study, differences in the use of biplane vs. out-of-plane ultrasound techniques can be explained [18]. It is possible that the smaller diameter of the peripheral vessels in Convissar's study made visualization and access more difficult, which could have affected the effectiveness of the out-of-plane technique and improved the effectiveness of the biplane technique. In contrast, in our study, the larger diameter of the central vessels may have allowed for easier visualization and access, which could have reduced the differences in the effectiveness of the ultrasound techniques used.

In the study by Scholten et al., where the same type of ultrasound machine was used to compare the biplane technique vs. in-plane and out-of-plane techniques, the choice between any of the 2D techniques was subject to the preference of the professional at the time of performing radial artery catheterization in patients undergoing elective cardiothoracic surgery. It was found that 90% of the professionals in the 2D technique group preferred the use of the out-of-plane vs. in-plane technique [19]. In terms of overall success and success in the first attempt, as well as in the posterior wall puncture, no significant differences were found. No difference was observed in secondary outcomes such as the number of punctures, total procedure time, and perceived mental effort during the procedure by the operator.

In the study by Jones et al., where the biplane vs. out-of-plane technique was compared for the insertion of an internal jugular catheter in a simulated model, a procedure performed by emergency medicine residents with previous experience in the use of ultrasound guidance for obtaining vascular access, no significant differences were found in the success of cannulation or the frequency of puncture of the posterior wall [20]. Regarding secondary outcomes, a significant difference was observed in the time required for cannulation, being shorter in out-of-plane than in biplane. Concerning the success of cannulation on the first attempt or puncture of the posterior wall, no significant differences were found between the techniques.

## Limitations

This study has some limitations that should be acknowledged. First, the study was conducted in a simulation model, and the results may not necessarily translate to real patients. Although the simulation model allowed for standardized conditions, it is important to note that the anatomy and physiology of a simulated model may differ from that of a real patient. Therefore, caution should be exercised when interpreting the results of this study and further research should be conducted in real patient populations.

Second, all procedures were performed by emergency physicians with varying degrees of experience. While this reflects the current clinical practice, it may limit the generalizability of the results to other specialties or levels of experience. Future studies should include physicians from different specialties and levels of experience to better understand the applicability of these techniques in various clinical settings.

Despite these limitations, this study provides valuable insights into the efficacy of different ultrasound techniques for vascular access in a simulated model. Further research is needed to confirm these findings in real patient populations with a wider range of healthcare providers.

## Conclusions

In this simulated model experiment for ultrasound-guided jugular vascular access with pocket ultrasound operated by emergency medicine physicians, no significant differences were evident when comparing biplane visualization with in-plane or out-of-plane visualization techniques in terms of cannulation success, time required for access, need for needle redirection, or frequency of puncture of the posterior vessel wall. The study results show that all three ultrasound planes performed well not only in terms of success but also in safety, which is important given their much lower cost compared to conventional equipment. These findings have important implications for clinical practice and highlight the potential benefits of using pocket ultrasound machines for internal jugular vein cannulation.

## Data Availability

The data stored digitally on the university servers provided for this and are available from the corresponding author on reasonable request.
